# A new approach to measuring individual differences in sensitivity to facial expressions: influence of temperamental shyness and sociability

**DOI:** 10.3389/fpsyg.2014.00026

**Published:** 2014-02-03

**Authors:** Xiaoqing Gao, Julia Chiesa, Daphne Maurer, Louis A. Schmidt

**Affiliations:** ^1^Centre for Vision Research, York University, Toronto, ONCanada; ^2^Department of Psychology, Neuroscience & Behaviour, McMaster University, Hamilton, ONCanada

**Keywords:** temperamental shyness, sociability, emotional facial expression, fear, hypervigilance, avoidance

## Abstract

To examine individual differences in adults’ sensitivity to facial expressions, we used a novel method that has proved revealing in studies of developmental change. Using static faces morphed to show different intensities of facial expressions, we calculated two measures: (1) the threshold to detect that a low intensity facial expression is different from neutral, and (2) accuracy in recognizing the specific facial expression in faces above the detection threshold. We conducted two experiments with young adult females varying in reported temperamental shyness and sociability – the former trait is known to influence the recognition of facial expressions during childhood. In both experiments, the measures had good split half reliability. Because shyness was significantly negatively correlated with sociability, we used partial correlations to examine the relation of each to sensitivity to facial expressions. Sociability was negatively related to threshold to detect fear (Experiment 1) and to misidentify fear as another expression or happy expressions as fear (Experiment 2). Both patterns are consistent with hypervigilance by less sociable individuals. Shyness was positively related to misidentification of fear as another emotion (Experiment 2), a pattern consistent with a history of avoidance. We discuss the advantages and limitations of this new approach for studying individual differences in sensitivity to facial expressions.

## INTRODUCTION

The accurate detection and identification of facial expressions of emotion is critical to optimal social interaction and human survival. Yet previous studies have shown a long developmental course for children’s recognition of facial expressions, especially the negative ones (Camras and Allison, 1985; Kolb et al., 1992; Markham and Adams, 1992; Gosselin and Larocque, 2000; De Sonneville et al., 2002; Mondloch et al., 2003; Durand et al., 2007; Thomas et al., 2007; Herba et al., 2008; Gao and Maurer, 2009, 2010; Montirosso et al., 2010). Although children do rapidly learn to recognize intense emotional expressions, the decoding of the more subtle expressions seen everyday is more difficult for them. This pattern was evident when we developed a new method for measuring sensitivity to subtle facial expressions (Gao and Maurer, 2009, 2010). Specifically, we measured children’s sensitivity to facial expressions with 20 levels of intensity spanning from neutral to intense expressions. We found in typically developing children, the threshold to detect happy facial expressions is adult-like by age 5. Children’s threshold to detect other facial expressions improves between age 5 and 10 (for sadness, fear, surprise, and disgust) or even after 10 (for anger). Misidentifications follow a different developmental trajectory, with some errors common only in childhood (e.g., fear mistaken as sadness).

As well as developmental changes in sensitivity to facial expressions, our previous studies documented considerable variance within each group, especially for negative expressions, and even in adults (Gao and Maurer, 2009, 2010). Here we investigated whether this methodology might be useful for studying individual differences. We did so by measuring the reliability of individual differences in our threshold and misidentification measures and their relation to individual differences in temperamental shyness.

Temperamental shyness appears to have its origins in early infant motor and affective behavior and is associated throughout development with a number of distinct psychophysiological correlates at rest and in response to social provocation, including greater relative right frontal EEG activity, high and stable heart rate, and high morning salivary cortisol responses (see Schmidt et al., 2005; Schmidt and Buss, 2010, for a review).

Shyness seems to be one logical individual difference factor to investigate, given that the origins and maintenance of shyness are linked to social interaction and social contexts. Shyness reflects heightened fear and inhibition in, and avoidance of, real or anticipated social situations (Cheek and Buss, 1981). Shyness is weakly to moderately correlated with the Eysenck neuroticism dimensions and moderately inversely correlated with the Eysenck extraversion dimension (Jones et al., 1986; Kamath and Kanekar, 1993; Rai, 2011). Factor analytic studies have consistently shown that a shyness factor is located in the space between the extraversion and neuroticism dimensions in personality measures in childhood and adolescence (Shiner and Caspi, 2003) and in adulthood (Crozier, 1979).

The distinctiveness of shyness as a psychological construct is that it refers specifically to insecurities in a social context. People withdraw from social situations for different reasons. Some retreat because they are inhibited in social situations (i.e., they are shy). Others retreat because they do not have a need to affiliate with others (i.e., they are introverted) or low in sociability. Eysenck distinguished between introverted social shyness (the preference for one’s own company but a capacity to function effectively in social situations) and neurotic social shyness characterized by self-consciousness and anxiety about social encounters (Eysenck, 1956; Eysenck and Eysenck, 1969). This distinction has been supported by studies that identified separate factors of lack of sociability and of shyness (Cheek and Buss, 1981; Jones et al., 1986; Bruch et al., 1989; Reviewed in Crozier, 2005).

Despite the fact that shyness seems to be an intuitively obvious personality style to examine, studies of face processing in shyness are limited. The studies that do exist on the topic primarily have used behavioral measures with children. These studies point to distinct behavioral correlates in children who are shy. For example, shyer children are more likely to make errors in recognizing photographs of facial expressions (Simonian et al., 2001; Battaglia et al., 2004, 2005) and in discriminating faces based on the spacing of the features (Brunet et al., 2010). They also tend to dwell more on the eyes and less on the mouth when processing unfamiliar faces than their less shy peers (Brunet et al., 2009).

Recent studies of shyness and face processing in adults have focused primarily on neural correlates. For example, measures of event-related EEG potentials (ERP) indicate that, compared to non-shy adults, shy adults exhibit an increased latency and reduced amplitude of the first positive deflection (the P1 wave) when viewing fearful faces (Jetha et al., 2012). Measures of functional Magnetic Resonance Imaging indicate greater bilateral amygdala activation (Beaton et al., 2008) and reduced fusiform activity (Beaton et al., 2009) during the processing of unfamiliar faces by shy adults. Some of these neural patterns match a vigilance hypothesis in which shy individuals appear to be initially vigilant to faces that are potentially threatening in the case of unfamiliar faces (as indicated by the heightened amygdala activation). Other neural patterns match an avoidance hypothesis, which could be a way of regulating heightened emotion (as indicated by the reduced fusiform activity for unfamiliar faces and reduced ERP response for fearful faces).

In the current study, we used well established and validated measures of temperament and face stimuli varying in intensity within emotion category. Our purpose was to evaluate whether our novel method is useful for studying individual differences in temperament and at the same time to advance knowledge of the impact of temperamental shyness on processing of facial expressions. We measured shyness with the Cheek and Buss Shyness and Sociability scale (Cheek and Buss, 1981), a widely used measure in the adult personality literature (see Schmidt and Buss, 2010). The scale has good internal consistency [coefficient alpha = 0.82 (Bruch et al., 1989)] and test–retest reliability [Chronbach’s alpha = 0.79 (Cheek and Buss, 1981)]. Another part of the scale measures an orthogonal temperamental dimension: sociability. Independent of shyness, sociability relates to how frequently one seeks social encounters and opportunities, or the inverse that is more relevant here, how frequently one avoids social encounters (Cheek and Buss, 1981).

We related the individual scores on shyness and sociability to our measures of sensitivity to facial expressions. Specifically, we conducted two separate experiments to examine the relation of shyness and sociability to sensitivity to facial expressions in non-clinical samples of female university students. Accuracy is widely used as a conventional measure of sensitivity to intense facial expressions. However, with subtle expressions – of the type seen in everyday interactions – accuracy is not informative because it does not distinguish failure to see that the face is expressing an emotion from correct recognition that the face is not neutral but inability to accurately identify the emotion being expressed. As in our studies of normal development (Gao and Maurer, 2009, 2010), we used two measures of sensitivity to facial expressions: (1) the thresholds to detect low intensity facial expressions as distinct from neutral, and (2) accuracy in recognizing which facial expression was present in the face above that detection threshold. We hypothesized that if being shy and/or socially avoidant during childhood leads to decreased exposure to facial expressions, adults with that history would make more misidentifications (e.g., lower accuracy) than typical adults. If, on the other hand, shy, socially avoidant individuals are hypervigilant to signs of threat, then we expect adults with that history to have heightened sensitivity (e.g., lower thresholds). It is also possible that these two factors may work together to affect the final outcomes.

In Experiment 1, we collected sensitivity measures for expressions of happiness, sadness, fear, and disgust. In Experiment 2, we collected data for expressions of happiness, sadness, fear, and anger in order to replicate the results from Experiment 1 and to extend the findings to the processing of threatening expressions, namely anger. In both experiments, we evaluated the reliability of our two expression measures and their relation to measures of shyness and sociability.

## EXPERIMENT 1

### METHODS

#### Participants

Forty-one female undergraduate students (18–28 years old, *M*_age_ = 18.0 years) participated in Experiment 1. The participants received either credit for an introductory psychology course or $10 (Canadian) compensation for their time. All the participants were Caucasian and had normal or corrected-to-normal vision. An additional six participants were excluded from the final sample because they were not Caucasian (to avoid potential cultural confounding since Caucasian faces were used as stimuli, *n* = 4) or failed visual screening (*n* = 2; criterion for passing: Snellen acuity of 20/20 or better in each eye).

#### Affective face stimuli

**FIGURE 1:**
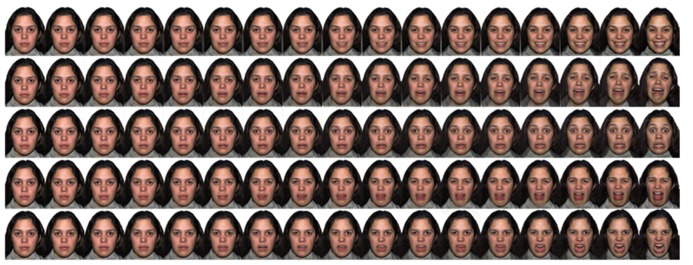
**A sample of facial expressions with varying intensity levels**.

We selected photographs of four models (two females), each posing facial expressions of happiness, sadness, fear, and disgust plus neutral from the NimStim face database (Tottenham et al., 2009). The images represent either no expression (neutral) or intense expressions of the designate emotions that had been recognized with high accuracy by adults in a previous validation study (Palermo and Coltheart, 2004). We created intermediate intensities of expressions by morphing each intense emotional face with its corresponding neutral face of the same model (for details, see Gao and Maurer, 2009). For each model and facial expression, we created 16 levels of intensity in 5% steps for the range from 5 to 55% and 10% steps for the range from 60 to 100% (**Figure 1**)^1^. We used larger steps (thereby fewer levels) for the relatively high intensity expressions because previous studies with the same stimulus set show that adults’ accuracy for recognizing these facial expressions reaches an asymptote when the intensity is above 60% (Gao and Maurer, 2009, 2010). In total, there were 260 expressions comprised of 4 expressions × 16 intensities × 4 models and 1 neutral expression × 4 models. The images were printed individually on 4 × 6^′′^ photo paper in full color with lamination using an inkjet printer at 300 dpi. The size of the pictures was approximately 7 cm (width) × 11 cm (height).

#### Procedures and stimuli

Upon arrival, we described the procedures to the participant and obtained written consent.

**FIGURE 2:**
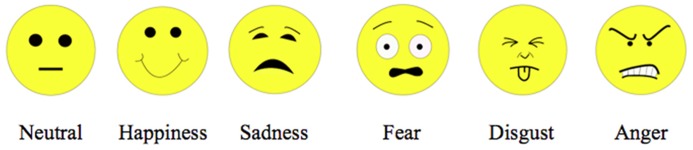
**Emotion icons**.

The procedure was the same as in our previous studies of normal development (Gao and Maurer, 2009, 2010) and hence took the form of a child-friendly story^2^. We introduced the participants to a game in which they were to help people to find the right house based on people’s feeling. Five miniature houses with emotion icons (**Figure 2**) representing happiness, sadness, fear, disgust, and neutral attached to the roofs were presented on a table in front of the participant. The emotions represented by the icons were accurately recognized by another group of adults in a pilot study. On each trial, the participant was handed one picture and, based on which emotion the person in the picture was perceived to be feeling, put the picture into a slot in the roof of the house with the corresponding emotion icon. The experimenter could not see the stimuli the participant was judging on each trial. The slots in the roofs were narrow (1 cm wide) so that the participant could not see the pictures already placed in the miniature houses. Each participant judged 136 pictures consisting of all four expressions at varying intensities, plus neutral of one randomly chosen female model and one randomly chosen male model (4 expressions × 16 intensities × 2 models + 4 neutral face × 2 models = 136 images). Four copies of the neutral face of each model were included so that the number of pictures put into the expressive houses would not be substantially larger than the number placed into the neutral house.

The protocol was approved by the McMaster Research Ethics Board.

#### Temperament measures

After finishing the facial expression task, the participants filled out the Cheek and Buss Shyness and Sociability scale (Cheek and Buss, 1981). It comprises two subscales formed from the five items with the highest loading (Bruch et al., 1989) from the original shyness subscale (e.g., I find it hard to talk to strangers.), and the original five-item sociability subscale (Cheek and Buss, 1981; e.g., I like to be with people.). Participants rated each item on a five-point Likert scale, ranging form “not at all characteristic” (0) to “extremely characteristic” (4), giving a possible range of 0–20. Recent data (Rai, 2011) from a sample of young adults (*n* = 152) found that Shyness and Sociability scores were weakly to moderately correlated with the Eysenck personality subscales of extraversion (shyness, -0.67; sociability, 0.58) and neuroticism (shyness, 0.51; sociability, -0.25). To assess the discriminant validity of the shyness and sociability subscales, we corrected the above correlation coefficients with the reported reliability scores of the four constructs (Shyness: 0.82, sociability: 0.76, Bruch et al., 1989; Extraversion: 0.82, Neuroticism: 0.83, Caruso et al., 2001). After the correction, the correlation values were -0.82 between shyness and extraversion, 0.73 between sociability and extraversion, 0.61 between shyness and neuroticism, and -0.31 between sociability and neuroticism. According to a criterion suggested by previous researchers (Ten Berge, 1986; Cooke and Michie, 2001), if the corrected correlation coefficient is smaller than 0.85, the discriminant validity is considered as acceptable. Therefore, it is reasonable to consider shyness and sociability as different constructs from extraversion and neuroticism.

Although theoretically the shyness and sociability scores are thought to be orthogonal, in fact they are often correlated negatively in studies of young adults (Cheek and Buss, 1981; Bruch et al., 1989; Schmidt and Fox, 1994; Schmidt, 1999; Miller et al., 2008; Young and Brunet, 2011). Therefore, we checked for the correlation in the current sample, and corrected the subsequent analyses for that correlation by reporting partial correlations.

#### Data analysis

As in previous studies (Gao and Maurer, 2009, 2010), for each facial expression, we identified two types of errors: (a) putting an emotional face in the neutral category when the intensity was low, and (b) putting one facial expression in the wrong emotion category (e.g., misidentifying fear as sadness) when intensity was higher. To measure these two types of sensitivity, we calculated (a) the threshold to discriminate each expression as different from neutral, and (b) the rates of misidentification when the faces were above threshold, that is, classified as non-neutral. Unlike our separate measures, an analysis of accuracy will confound the two types of error: for example, seeing a fearful face as neutral and mistaking a fearful face as sad. In each case, we averaged across the participant’s independently derived values for the two models.

***Thresholds.*** Because we included low intensity facial expressions in the current experiment, it is possible that people may fail to see any emotion in a face if the intensity is below a threshold level. Here we defined the threshold level as the intensity level where 50% of the time an emotional face is classified as neutral. To calculate threshold, we coded the responses as 0 (neutral) or 1 (non-neutral), with the non-neutral responses including all the responses classifying a face as showing any emotion (both the correct emotion categories and the wrong emotion categories). We assumed that as the intensity increases, the probability that a face would be classified as showing emotion (non-neutral) would increase. Such probability would reach 1 when the expression is highly intense. Therefore, we estimated the threshold value by fitting a cumulative Gaussian function to the data of each participant using the following formula:

(1) P(identification)=1σ2π∫−∞x     exp⁡(−(u−μ)22σ2)du⁢

where *x* is intensity level and *P* is the probability of identifying a face as showing emotion. The two parameters, μ and σ, are the mean and standard deviation, respectively, of the normal distribution *X* ~ *N*(μ, σ^2^). We estimated μ using a maximum likelihood procedure, which gave us the best estimate of μ while minimizing the influence of σ on the estimation. With the estimated best fitting cumulative Gaussian function, we calculated the threshold as the intensity level that corresponds to 50% probability on the cumulative Gaussian function. The threshold measure shows how sensitive a person is in detecting emotion from low intensity facial expressions.

***Misidentification rates.*** When a face is classified as showing emotion (i.e., non-neutral), besides the correct classification, people may make errors by classifying a face into a wrong emotion category. To measure misidentification rates, we calculated the proportion of the wrong responses out of the total number of non-neutral responses for each expression. Different from the threshold measure, misidentification rates show how often a person confuses faces coming from different emotion categories.

***Reliability of the measures.*** To assess the reliability of the threshold and misidentification rate measures, we split the raw data for each expression into two halves and calculated Cronbach’s alphas between independently derived estimates from the two halves. One half of the split data had eight levels of intensity including 5, 15, 25, 35, 45, 55, 70, and 90%. The other half of the split data had the remaining eight levels of intensity including 10, 20, 30, 40, 50, 60, 80, and 100%. We summarized the reliability score (Chronbach’s alpha) of each measure in **Table 1**. Reliability scores ranged from 0.65 to 0.86 (mean = 0.77), suggesting that the current measures are reliable and hence suitable for analyses of individual differences.

**Table 1:** Descriptive statistics of expression sensitivity measures and their correlation with temperament in Experiment 1.

Sensitivity to facial expressions	*N*^a^	Mean	Range	Alpha	Correlation^b^	
					Shyness	Sociability
**Threshold**
Happy	41	10.9	5–27.5	0.86	-0.06 (-0.08)	0.04 (0.06)
Sad	41	19.9	5–40.0	0.74	-0.01 (-0.08)	0.18 (0.19)
Fear	41	12.6	5–27.5	0.82	0.09 (-0.01)	0.29+ (0.27)
Disgust	41	13.6	5–27.5	0.72	0.05 (0.00)	0.15 (0.15)
**Misidentification**
Happy	41	0.04	0–0.19	0.65	0.22 (0.23)	0.01 (-0.08)
Sad	40	0.30	0–0.74	0.84	0.10 (0.12)	-0.05 (-0.09)
Fear	41	0.15	0–0.47	0.72	0.06 (0.12)	-0.16 (-0.20)
Disgust	41	0.21	0–0.55	0.80	-0.01 (0.02)	-0.08 (-0.08)

*+*p* = 0.066.*

a The sample size for the correlation analysis with outliers removed.

b Partial correlations controlling for the correlation between shyness and sociability. Zero-order correlations are in parentheses.

***Correlation between temperament and sensitivity to facial expressions.*** In the current sample, there was a good range of scores on the shyness measure (0 to 17 out of a possible 20 points, mean = 7.6, SD = 3.5) and sociability measure (10 to 18 out of a possible 20 points, mean = 15, SD = 2.5), with enough variability in the sample to evaluate whether shyness and sociability were related to sensitivity to facial expressions. As has been found in previous studies of non-clinical populations (Cheek and Buss, 1981; Bruch et al., 1989; Schmidt and Fox, 1994; Schmidt, 1999; Miller et al., 2008; Young and Brunet, 2011), the shyness scores were negatively correlated with sociability scores (r = -0.35, p < 0.01): individuals who scored high on the shyness subscale tended to have low sociability scores. To control for the correlation between the shyness and sociability measures, we calculated partial correlations between shyness and measures of sensitivity to facial expressions controlling for sociability, and calculated partial correlations between sociability and measures of sensitivity to facial expressions controlling for shyness. We identified outliers in the measures of sensitivity to facial expressions with a three standard deviation criterion, and removed the outliers for each emotion category before the correlation analysis. To assess the significance of the correlations, we converted correlation coefficients (*r*) to *t* values using the following formula and tested the null hypothesis that the *r* values are not different from 0 (two-tailed test):

(2) $$t=r\,\sqrt{\frac{n-2}{1-r^{2}}}\,$$

where *r* is the correlation coefficient and *n* is the sample size.

### RESULTS AND DISCUSSION

In the current sample, our measures of both threshold and misidentification showed good split half reliability and a range of values that might reflect stable individual differences. However, there was little relation to temperament. The measure of shyness was not correlated with any of the measures of sensitivity to facial expression, once we removed the effect of its (negative) correlation with sociability. Sociability, on the other hand, was marginally significantly correlated with threshold to detect emotion in fearful faces (*r* = 0.29, *p* = 0.066), suggesting that less sociable individuals were more likely to detect emotion in fearful faces. No other correlation was significant for any other emotion.

The elevated sensitivity to fear expressions in less sociable adults is consistent with the hypervigilant hypothesis that less sociable individuals are hypersensitive to threat and other socially relevant cues (Garner et al., 2006; Brunet et al., 2009; Jetha et al., 2012). Importantly, the relation between social avoidance and hypervigilance to fearful emotions was revealed in the responses to low intensity facial expressions such as those most likely encountered in everyday interaction. If we had used only intense expressions, like most previous studies, we might not have detected the subtle individual differences reported here.

## EXPERIMENT 2

In Experiment 2, we tested a different group of female adults with a new combination of expressions (happiness, sadness, fear, and anger) in order to test the reliability of the findings in Experiment 1, with an overlapping set of expressions (happiness, sadness, fear), and at the same time explore the relation between temperamental shyness and sociability with sensitivity to angry expressions. We included the emotional expression of anger because we expected it to be especially salient for people who are shy or socially avoidant even at low intensities, given that it is presumed to signal threat.

### METHOD

#### Participants

Forty-five female undergraduate students (17–28 years old, *M*_age_ = 20.1 years) participated in Experiment 2. The participants either received credit for an introductory psychology course or a $10 (Canadian) compensation for their time. All the participants were Caucasian and had normal or corrected-to-normal vision. An additional six participants were excluded from the final sample because they were not Caucasian (*n* = 3) or failed visual screening (*n* = 3).

#### Procedure and stimuli

We used the same stimuli as in Experiment 1, except that we replaced facial expressions of disgust with facial expressions of anger that were created in the same way. A different female experimenter tested all the participants in Experiment 2. In all other respects, the procedure was the same as in Experiment 1.

#### RESULTS AND DISCUSSION

The sample of females in Experiment 2 had a similar range of shyness scores to the group in Experiment 1 (0–16, mean = 7.0, SD = 4.3). The range of the sociability scores was wider in this sample than the previous sample (0–20, mean = 14.0, SD = 4.1), although the mean shyness score and sociability score did not differ between the two groups (*ps* > 0.5). We identified one outlier in the temperament measures who had a very low shyness score (2) and a very low sociability score (0), a pattern that was very different from the rest of the group. With this outlier removed, in the sample of females in Experiment 2, shyness scores were negatively correlated^3^ with sociability scores (*r* = -0.45, *p* < 0.01), as was found in Experiment 1. We also removed outliers in the measures of sensitivity to facial expressions using a three standard deviation criterion for each emotion category before running the correlation analyses. Because of the correlation between shyness and sociability, as in Experiment 1, we conducted partial correlations for the analysis of the relation between our measures of sensitivity to facial expressions and shyness and sociability.

We assessed the reliabilities of the measures of sensitivity to facial expressions with the same split half analysis as in Experiment 1. Except for the misidentification rates for anger, which had a relatively low split half reliability (0.59), all the other measures had reasonably high reliabilities (range = 0.66–0.86, mean = 0.77, **Table 2**), which were similar to what we found in Experiment 1.

**Table 2:** Descriptive statistics of expression sensitivity measures and their correlation with temperament in Experiment 2.

Sensitivity to facial expressions	N*^a^*	Mean	Range	Alpha	Correlation^b^with	
					Shyness	Sociability
**Threshold**
Happy	43	15.6	5–35.0	0.82	-0.09 (-0.07)	-0.08 (-0.04)
Sad	44	20.1	5–40.0	0.82	-0.02 (0.01)	-0.05 (-0.05)
Fear	44	15.0	5-32.5	0.79	-0.04 (-0.07)	0.06 (0.09)
Anger	44	15.5	5-30.0	0.66	-0.15 (-0.18)	0.02 (0.10)
**Misidentification**
Happy	42	0.02	0-0.16	0.86	0.13 (0.01)	0.29+ (0.26)
Sad	43	0.03	0-0.36	0.67	0.07 (-0.02)	0.19 (0.18)
Fear	43	0.13	0-0.36	0.78	0.32* (0.23)	0.27^#^ (0.15)
Anger	43	0.14	0-0.24	0.59	0.20 (0.12)	0.19 (0.12)

+p = 0.066; #p = 0.078; *p < 0.05.

a The sample size for the correlation analysis with outliers removed.

b Partial correlations controlling for the correlation between shyness and sociability. Zero-order correlations are in parentheses.

In the current sample, sociability scores tended to be positively correlated with misidentification rates for happy expressions (*r* = 0.29, *p* = 0.066). Less sociable people were less likely to make errors (were more accurate) with happy expressions. Further analysis on the type of misidentification participants made suggests that this effect was mainly driven by the confusion between happy expressions and fearful expressions, as sociability scores tended to be positively correlated with the frequency of happy faces being misidentified as fear (*r* = 0.32, *p* < 0.05), suggesting that less sociable people were less likely to make this type of confusion. There was no correlation with other types of misidentification for happy faces.

Both shyness and sociability tended to be positively correlated with misidentification rates for fear (*r* = 0.32, *p* < 0.05, *r* = 0.27, *p* = 0.078, for shyness and sociability, respectively). Since we calculated partial correlations, the relation between shyness and misidentification rates for fear and the relation between sociability and misidentification rates for fear were independent of each other. The correlation with shyness suggests shyer people were more likely to misidentify fearful expressions as showing another emotion. On the other hand, the correlation with sociability suggests less sociable people were less likely to confuse fearful expressions as another emotion. Further analysis on the types of confusion people made suggests fearful expressions in most of the cases were misidentified as sad, although the type of error did not vary systematically with either shyness or sociability scores.

The results from Experiment 2 provide further evidence that our measures of sensitivity to facial expression generate reliable differences among non-clinical populations of young adults that may be related to individual differences in temperament. The finding that less sociable adults were less likely to make errors about fearful expressions is consistent with the *hypervigilance* hypothesis for fearful expressions. They do not mistake happy expressions as fearful or misidentify fearful expressions as often as their more sociable peers: more sociable adults were more likely to misidentify happy expressions as fear and fearful expressions as another emotion: Surprisingly, we did not see the same relation for angry expressions, which were tested in Experiment 2 but not Experiment 1, perhaps because in this sample the expression measures were less reliable and/or because there was less variance for anger than for the fear measures. On the other hand, shyness was related to the tendency to confuse fear as another emotion. One possible explanation comes from the *avoidance* aspect of shyness. Avoidance, especially of faces perceived to be threatening, would lead to reduced experience with fearful expressions. This interpretation is consistent with evidence that shyer children scan faces differently from their non-shy peers and are poorer at recognizing intense facial expressions and subtle differences in identity (Simonian et al., 2001; Battaglia et al., 2004, 2005; Brunet et al., 2010). This relation may not have emerged in Experiment 1 because the foils with which fear could be confused were different.

## GENERAL DISCUSSION

We used a novel approach to measure individual differences in sensitivity to facial expressions. Using varying levels of intensity for each expression, we assessed two measures. The threshold measure revealed sensitivity to detect the presence of emotion in subtle facial expressions. The misidentification measure revealed confusions among different emotion categories. Our reliability analysis indicated that both measures were reliable. Both measures also produced the type of variability necessary to study individual differences in non-clinical samples of female adults.

We found individual differences in sensitivity to facial expressions as revealed with the current measures are related to temperamental shyness and sociability. In the first sample, less social females were more sensitive to detect emotion in fearful faces. In the second sample, less social females were less likely to mistake happy faces as showing fear, or to mistake fearful faces as portraying another emotion. Shyer females in the second sample were more likely to misidentify fearful faces.

The results for people low in sociability are consistent with a *hypervigilance* hypothesis (Garner et al., 2006), which suggests that socially avoidant and withdrawn individuals are hypersensitive to detecting threatening stimuli. In the current case, less sociable people showed heightened sensitivity to fearful facial expressions. They had lower detection threshold for fearful faces (Experiment 1) and were less likely to confuse fear with other expressions or happy expressions with fear (Experiment 2). However, we did not find similar relations for angry expressions in Experiment 2 (the only experiment in which they were tested), as one might expect from the hypervigilance hypothesis. We note here that in previous studies socially anxious and avoidant people have a greater tendency to interpret facial expressions as negative, including faster detection of angry faces (e.g., Miskovic and Schmidt, 2012).

The hypervigilance hypothesis does not completely explain the results for shyness. In Experiment 2, shyness was positively correlated with misidentification rates for fear, which was most commonly mistaken as sadness at all intensities. One possible explanation is that avoidance of threatening faces in shy individuals led to reduced experience with fearful expressions. Alternatively, or in addition, those whose social interactions are compromised by poor recognition of facial expressions might develop increased shyness as a coping strategy. However, this interpretation must be regarded as preliminary because the increased misidentifications of fear with increased shyness did not emerge in Experiment 1, and similar patterns did not hold for sad or angry expressions in either experiment.

Alternatively, the relations with temperament found here might be related to differences in scanning the eye region of faces, where the diagnostic information for fear has been shown to reside (Smith et al., 2005), and/or associated traits like social anxiety or depression (reviewed in Machado-de-Sousa et al., 2010). Future studies could investigate these possibilities by using eye tracking to measure eye movements while the judgments of facial expression are made and/or collecting measures of other traits in addition to temperament.

The current approach for measuring sensitivity to facial expressions yielded reliable variability (mean reliability score = 0.77). However, there are also limitations with the approach. First, we did not find consistent relations with temperament across the two samples. One possible reason is that we had different groupings of facial expressions in the two experiments: fear could be mistaken for disgust only in Experiment 1 and for anger only in Experiment 2. A future study could address this issue by testing participants with all five negative expressions, or even all six basic emotional expressions. It is not likely that the inconsistency across the two samples arose from poor reliability of our measures of sensitivities to facial expressions, because the split half reliability was reasonably high for both samples, with values for the measures correlated with temperament being especially high (0.82, 0.86, 0.78). However, it remains possible that the inconsistency arose from noise in the data, especially with the small sample size of the current study.

A second limitation is that we tested only two relatively small and highly homogeneous samples of non-clinical young female adults. In both of our samples, very few participants had shyness scores on the tails of the distribution, a pattern reflecting the distribution of shyness in the typical population. However, our typical distribution limited the power of the current study to detect any association between temperamental shyness and sensitivity to facial expressions and the relations we did find were weak and often only marginally significant. As for the measures of sensitivity to facial expressions, the threshold values were normally distributed. However, the misidentification rates were positively skewed, so that there were fewer cases with high misidentification rates than with low misidentification rates. Future studies could test a larger non-clinical population and focus only on individuals who fall on the tails of the distribution of the measures. Alternatively, it would be interesting to apply the current methodology to study sensitivity to subtle facial expressions in groups in whom more variance can be expected, namely clinical (e.g., anxiety disorder, depression) and child populations that vary in temperament.

A third limitation is that we used static faces with posed facial expressions. We note that the anchors (the high intensity expressions) were, nevertheless, recognized with high accuracy by typical adults in a previous validation from another laboratory (Palermo and Coltheart, 2004) and that the changes in intensity produced similar linear degrees of physical change across the expressions (Gao and Maurer, 2010). It also would be interesting for future studies to use dynamic and/or genuine facial expressions to investigate the individual differences in sensitivity to facial expressions with the measures described here.

Finally, previous studies have shown that shyness and sociability are correlated with extraversion and neuroticism. It would be important for future studies to investigate how individual differences revealed with the current measures of sensitivity to facial expressions are related to extraversion and neuroticism. The current conclusions are limited to females only. It would be interesting to test whether relations similar to those we found in the current study hold for shy and non-social males, as a female advantage has been reported in detecting emotions (Mayer et al., 2002; Brackett et al., 2006) and differences have been found in how shy males versus shy females process affective stimuli in child studies (Theall-Honey and Schmidt, 2006).

## Conflict of Interest Statement

The authors declare that the research was conducted in the absence of any commercial or financial relationships that could be construed as a potential conflict of interest.

## Footnotes

1 The morphed facial expression stimuli are available for research purposes upon request.

2 A computerized version of this task is available for research purposes upon request. Our pilot data show that the computerized version of the task yields the same results for adults without the child-friendly story.

3 The correlation remains significant even when the outlier is included.
